# Cerebrospinal Fluid Cytology in Lyme Neuroborreliosis Revisited—Role of Neutrophilic Granulocytes: A Retrospective Single-Center Study

**DOI:** 10.3390/jcm13237406

**Published:** 2024-12-05

**Authors:** Ferdinand Otto, Peter Wipfler, Wolfgang Hitzl, Martin Preisel, Andrea Harrer, Georg Pilz

**Affiliations:** 1 Department of Neurology, Christian-Doppler University Hospital, Paracelsus Medical University, Centre for Cognitive Neuroscience, Ignaz Harrer Str. 79, 5020 Salzburg, Austria; p.wipfler@salk.at (P.W.); georg.pilz@salk.at (G.P.); a.harrer@salk.at (A.H.); 2 Department of Neurology, University Hospital Zurich, University of Zurich, 8091 Zürich, Switzerland; 3 Research Office, Biostatistics, Paracelsus Medical University, 5020 Salzburg, Austria; wolfgang.hitzl@pmu.ac.at; 4 Department of Pediatrics, Paracelsus Medical University, 5020 Salzburg, Austria; m.preisel@salk.at; 5 Department of Dermatology and Allergology, Paracelsus Medical University, 5020 Salzburg, Austria

**Keywords:** Lyme neuroborreliosis, cerebrospinal fluid, chemokine cxcl13, granulocytes

## Abstract

**Background/Objectives**: diagnosis of Lyme neuroborreliosis (LNB) relies on medical history, clinical findings, and detection of pathogen-specific antibodies in the blood and cerebrospinal fluid (CSF). The chemoattractant CXCL13 serves as an additional marker for LNB acuity. During the diagnostic workup, cytomorphological examination of immune cells in CSF provides early insights. Lympho-monocytic pleocytosis with plasma cells and activated lymphocytes is usually described as a typical feature of LNB. In contrast we frequently observe a cytological cell picture featuring neutrophilic granulocytes as well as activated mononuclear cells and plasma cells in patients with LNB, which we refer to as a mixed cell picture. We, hence, investigated the presence of granulocytes to determine their role as typical findings associated with LNB. **Methods**: we conducted a retrospective analysis of CSF cytology in patients diagnosed with definite LNB at the Department of Neurology, Christian Doppler Medical Centre, Salzburg between 2015 and 2021. CSF results of patients with more than 10 erythrocytes/µL were excluded to avoid the presence of granulocytes due to artificial blood contamination. Additionally, CXCL13 levels were recorded, where available. **Results**: a total of 75 patients (42 female; 56%) met the diagnostic criteria of definite LNB. Cytology revealed the presence of granulocytes in the CSF of 91% of the patients (68/75). CXCL13 elevation was found to be significantly associated with the presence of granulocytes in CSF (*p* = 0.0025, or 1.009 (95% CI: 1.003–1.016). **Conclusions**: we confirm a mixed cell picture with granulocytes, activated mononuclear cells and plasma cells being a typical finding in the CSF cytology of LNB. The association between granulocytes and elevated CXCL13 suggests that their presence is a specific feature of the acute, untreated phase of LNB.

## 1. Introduction

About 200,000 patients in Europe and about 470,000 patients in the USA are affected by Lyme borreliosis each year [1,2]. Lyme neuroborreliosis (LNB) is the neurological manifestation of the systemic infection caused by the spirochete *Borrelia burgdorferi* sensu lato complex [3]. The neurological manifestation of Lyme borreliosis is reported in about 3–15% of the cases and may present as painful cranial neuritis, polyradiculitis, meningitis, or even, rarely, encephalomyelitis [4,5,6]. Diagnosis of LNB is established based on a combination of medical history, clinical findings, and laboratory tests for pathogen-specific antibodies in the blood and cerebrospinal fluid (CSF). Depending on the clinical manifestation and available laboratory results, the diagnosis of LNB can be further subclassified as possible, probable, and definite. The diagnosis of possible LNB is characterized by the typical clinical picture with detection of *Borrelia*-specific immunoglobulin (Ig)G and/or IgM in the serum but without available CSF data [7]. The diagnosis of probable LNB is based on the fulfilment of all the criteria of possible LNB as well as additional, non-specific inflammatory CSF findings, such as lymphocytic pleocytosis, blood–CSF barrier dysfunction, and intrathecal immunoglobulin synthesis. More precisely, intrathecal IgM synthesis occurs in 80–100% of LNB cases, and IgG synthesis in about 60% [3]. Intrathecal IgA appears to be more common in later stages of LNB [8]. In addition to the criteria of probable LNB, definite LNB requires the detection of intrathecally produced *Borrelia*-specific antibodies through positive IgG and/or IgM antibody index (AI) in the CSF, positive culture, or nucleic acid detection [4]. The AI is important to distinguish intrathecally produced antibodies from blood-derived antibodies which diffuse into the CSF via an impaired blood–CSF barrier (bCSFb). The AI represents the quotient of pathogen-specific IgG in the CSF and serum in relation to the quotient of total IgG in the CSF and serum [9].

The chemoattractant CXCL13 has been identified as a potent chemoattractant for B- lymphocytes, and CXCL13 elevations in CSF, additionally, have been proposed as both a diagnostic and a therapeutic response marker in patients with LNB [10]. However, CXCL13 has been found to be elevated in patients suffering from several other inflammatory diseases of the central nervous system (CNS) [11,12]. Notably, a decrease in CXCL13 levels within two weeks following antibiotic treatment supports its role as a potential marker for treatment response in patients with LNB [10,12]. Furthermore, intrathecal CXCL13 is an acuity marker and may serve as an additional biomarker for the diagnosis of acute LNB, particularly in clinical situations where antibody indices might still be negative [3,10,12,13].

Prior to the confirmation of a definite LNB diagnosis, CSF cytology provides early insights into the immune cell composition and can be informative regarding the etiology of the infection. Literature and consensus guidelines claim that CSF lympho-monocytic pleocytosis with plasma cells and activated lymphocytes is a typical feature of LNB [4,7].

Over several years of routine CSF cytomorphological examination, we have observed a cell picture featuring neutrophilic granulocytes, activated mononuclear cells, and plasma cells frequently associated with LNB, a phenomenon to which we refer as mixed cell picture. As this observation contrasts with the existing literature, we set out to address this discrepancy and retrospectively analyzed CSF cytologies from patients diagnosed with LNB between 2015 and 2021.

## 2. Material and Methods

### 2.1. Patients

We conducted a retrospective analysis of the medical records of all patients suspected to be suffering from LNB who had undergone lumbar puncture or CSF analysis at the Department of Neurology, Christian Doppler Medical Centre in Salzburg between 2015 and 2021. The study was approved by the local ethics committee of the Bundesland Salzburg (EK: 1056/2024 and 1066/2024); informed consent was waived due to the retrospective study design based on data retrievable from chart records. Only patients with definite LNB were included in the final analysis, with the diagnosis confirmed by presence/absence of intrathecal *Borrelia-burgdorferi*-specific antibody synthesis (positive IgG AI index, western blot, or positive *Borrelia* polymerase chain reaction (PCR) in the CSF).

CSF parameters specifically retrieved from medical records for this study included CSF cell count, CSF erythrocytes, cytological findings, and, if available, intrathecal CXCL13 levels. Intrathecal CXCL13 levels were measured using a standardized ELISA (Euroimmune, Lübeck, Germany). The tissue trauma of a lumbar puncture can contaminate the CSF with peripheral blood and falsify the result. Since in the peripheral blood the ratio of erythrocytes to white blood cells approximates 1000:1 [14], we referred to 10 erythrocytes per µL as the threshold for artificial blood contamination. The probability of finding a blood-derived granulocyte in the CSF was thus reduced to less than 1%. CSF laboratory diagnostics, including CSF cell processing, was performed by the Department of Laboratory Medicine at the Paracelsus Medical University, Salzburg, certified by the German Society for CSF Diagnostics and Clinical Neurochemistry (DGLN). We also collected demographic and clinical data including age, gender, timespan from the clinical onset to the lumbar puncture, and antibiotic treatment prior to the lumbar puncture.

### 2.2. CSF Cytology

**Figure 1:**
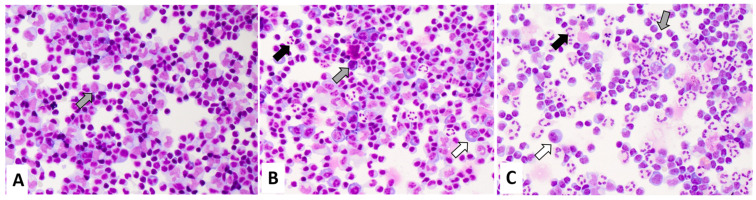
Exemplary May Grunwald Giemsa stainings of definite Lyme neuroborreliosis. The samples show activated mononuclear cells (grey arrow) and plasma cells (white arrow). The semi quantitative classification of granulocytes (black arrow) is displayed: (**A**) “no granulocytes” (**B**) “few granulocytes” (**C**) “several granulocytes”.

The cytomorphological evaluation of CSF immune cells was performed from GIEMSA-stained cytospin preparations by an experienced team of neurologists. Routinely, the presence of CSF immune cells (i.e., lymphocytes, monocytes, their activation states, presence of granulocytes and plasma cells) is semi-quantitatively categorized as few, several, or abundant. Cell types not mentioned (i.e., granulocytes, plasma cells in an inflammatory setting) are classified as not present. The semi-quantitative presence of neutrophilic granulocytes is further assessed in relation to mononuclear cells (i.e., lymphocytes and monocytes) and categorized as few granulocytes (fewer neutrophilic granulocytes than mononuclear cells), several granulocytes (more than a few neutrophilic granulocytes, with approximately comparable numbers of neutrophilic granulocytes and mononuclear cells), and abundant granulocytes (more neutrophilic granulocytes than mononuclear cells). For this study, we also included the category “no granulocytes”. Similarly, the presence of plasma cells was dichotomized into no plasma cells and plasma cells. An example of the cytological CSF classification is shown in Figure 1.

### 2.3. Statistical Methods

Data were checked for consistency and normality by using the Shapiro–Wilk test. Fisher’s exact test and Pearson’s chi-squared test were used to analyze cross tabulation tables. A one-factorial ANOVA, with corresponding LSD tests, was used for paired post-hoc comparisons. Univariate logistic regression models were used to analyze associations between the timing of the lumbar puncture post symptom onset, CXCL13 presence, and CSF cell count, on one hand, and the occurrence of neutrophilic granulocytes, on the other. Corresponding odds ratios with 95% CI were computed. All reported tests were two-sided, and *p*-values < 0.05 were considered statistically significant. All statistical analyses in this report were performed using STATISTICA 13 (Hill, T. & Lewicki, P. Statistics: Methods and Applications. StatSoft, Tulsa, OK, USA).

## 3. Results

An initial screening identified 88 patients with suspected LNB. Data clearing led to the exclusion of 13 patients (duplicate records (*n* = 2), missing clinical information (*n* = 2), did not meet diagnostic criteria *n* = 9)). Seventy-five patients (42 female; 56%), with a median age of 59 years (range: 2–82 years), met the diagnostic criteria for definite LNB. The median time from the reported symptom onset to the lumbar puncture was 14 days (range: 1–336 days). Elevated CSF cell counts were present in all patients, with a median CSF cell count of 92 cells/µL (range: 8–725 cells/µL). The median *Borrelia*-specific IgG AI of 8.3 was determined from 66 patients (range: 1.5–177). In the remaining nine (12%) patients, the *Borrelia*-specific AI was not determined. Definite LNB was established by PCR in four patients, with detection of *Borrelia*-specific bands in CSF by western blot in five patients. In 62 (83%) patients, intrathecal CXCL13 levels could be retrieved (median: 487 pg/mL; range: 60–710 pg/mL). The cohort’s descriptive parameters are displayed in Table 1.

**Figure 2:**
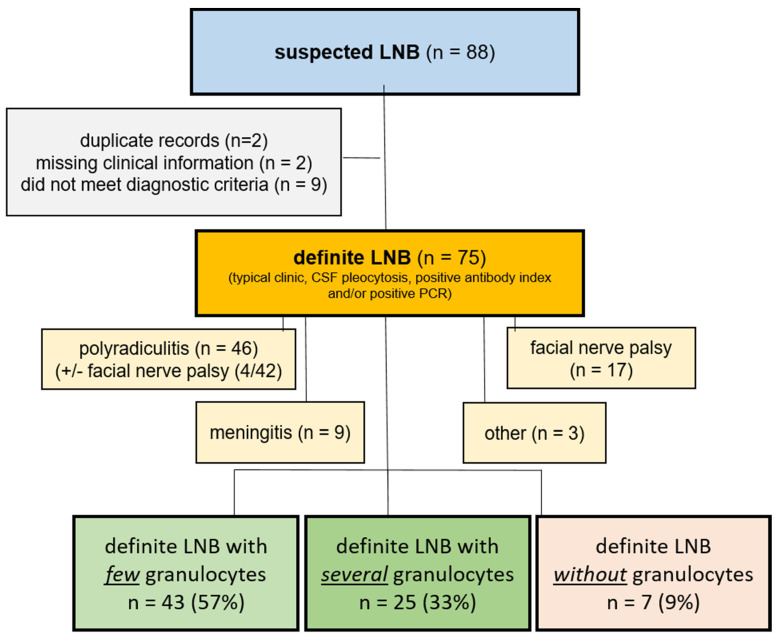
Flow chart for inclusion of patients. Initial screening resulted in suspected LNB (blue box). Data clearing (gray box) resulted in patients with definite LNB (yellow box). Clinical features of patients with definitive LNB are displayed (light yellow boxes). Percentages of neutrophilic granulocytes are shown (green boxes, red box) CSF: cerebrospinal fluid; LNB: Lyme neuroborreliosis; PCR: polymerase chain reaction.

In 91% of the patients (68/75), granulocytes were present in the cytological specimen report of the CSF: few granulocytes were reported in 57% (43/75) of the patients, several granulocytes were reported in 33% (25/75) of the patients, and no granulocytes were reported in 9% of the patients (7/75). We did not observe any cases with abundant granulocytes. Plasma cells were present in 65% (49/75) of all cases. In the cases with few or several granulocytes, plasma cells were present in 63% (*n* = 27/43) and 72% (*n* = 18/25) of the patients, respectively. In 57% (*n* = 4/7) of the cases with no granulocytes, plasma cells were present. Figure 2 shows a flow chart for the inclusion of the patients and the percentages of the subgroups.

With granulocytes present in the vast majority of cases, we next set out to investigate whether their occurrence or absence was associated to a specific clinical disease manifestation of LNB. The clinical presentation of patients with definite LNB is shown in Table 2. The most common clinical presentation was with polyradiculitis (46/75, 61%), followed by facial nerve palsy (17/75, 23%), and meningitis (9/75, 12%). Occurrence of neutrophilic granulocytes was similar in all groups.

We next investigated the existence of a potential correlation between the time from the reported symptom onset to the lumbar puncture, CSF cell count, and CXCL13 level elevations, on one hand, and the occurrence of granulocytes, on the other. We found no association with the time of the reported onset (r = −0.11, *p* = 0.37) and CSF pleocytosis (odds ratio (OR) = 1.0 (95% CI: 0.99–1.01, *p* = 0.68). CSF CXCL13 was significantly associated with the occurrence of granulocytes (*p* = 0.0025, OR 1.009 (95% CI: 1.003–1.016)).

With regard to antibiotic pre-treatment, granulocytes were less frequently observed in patients receiving an antibiotic pre-treatment prior to the diagnostic lumbar puncture. Granulocytes occurred in the CSF of six of the nine patients (67%) subject to antibiotic therapy and in 94% (48/51) of the patients not subject to antibiotic therapy (*p* = 0.038, OR = 8.0 (95% CI: 1.31–48.9)).

## 4. Discussion

There is a strong consensus on the inclusion of CSF cytology in the laboratory diagnostic pathway of acute LNB [3,4,8]. In contrast to our observations, the composition of immune cells in the CSF cytology of patients with acute LNB is widely suggested to be mononuclear.

Our retrospective, single-center analysis of patients with definite LNB, diagnosed between 2015 and 2021, confirms our observations from routine CSF cytology. Occurrence of granulocytes in patients with LNB is not the exception, but rather the rule. This observation challenges current diagnostic guidelines on LNB which only describe the presence of lymphomonocytic cells and plasma cells [4]. However, published evidence on CSF cytology findings in individuals suffering from LNB is sparse, with few authors mentioning granulocytes, with these often found in an incidental manner [15,16,17,18]. In 1987, Pohl et al. described the observation of a combination of lymphocytes, monocytes, and plasma cells as a typical cytological finding in the CSF. Notably, granulocytes are not mentioned in their publication [19]. Shah et al., for instance, aimed to differentiate LNB from enteroviral meningitis in children. They observed lower CSF granulocyte counts in the patients suffering from LNB, a phenomenon which they believed might be the result of children with LNB presenting symptoms later in the course of the disease than those with enteroviral meningitis [17]. Similarly, two further studies [16,18] investigating children with LNB reported a frequency distribution of CSF granulocytes of roughly 1%. These results, however, should be viewed critically, as the use of automated cell counting and differentiation is not considered to be reliable for investigating CSF cells [7].

Our cohort mainly comprised adults of an advanced age, and we cannot support the hypothesis of Shah et al. regarding a temporal relation between the time period from the reported symptom onset to the lumbar puncture and the occurrence of CSF granulocytes. Rather, the correlation between granulocyte presence and CXCL13 in the CSF suggests that the presence of granulocytes is a particularly characteristic feature of the acute, untreated phase of LNB.

Prescription of an antibiotic treatment (ceftriaxone or doxycylcin) is the established standard when treating LNB and leads to a decrease in CSF pleocytosis, antibody index, intrathecal CXCL13, and normalization of the CSF cell composition [7,10,20,21]. We additionally observed that the antibiotic treatment decreased the prevalence of granulocytes in the CSF of patients with acute LNB.

Given that the majority of LNB cases in our cohort showed elevated CXCL13 levels at a median time from the symptom onset to the lumbar puncture of 14 days, it is reasonable to assume that granulocyte appearance in patients with LNB reflects the acute phase of the disease. Beyond our observations, this hypothesis is supported by the case report of Borde et al. which describes a patient with LNB featuring marked CSF pleocytosis, an initial CSF granulocyte count of 70%, and very high levels of intrathecal CXCL13 [22]. At the follow-up, 12 days after ceftriaxone prescription, CSF granulocytes had disappeared and CXCL13 levels had significantly decreased. The authors proposed that the occurrence of granulocytes could be related to early aseptic meningitis. We consider this to be plausible, as neutrophilic pleocytosis, defined as a CSF neutrophil presence of more than 50%, may also be present in the early phases of viral meningitis [23]. Granulocytes may play a similar role in acute LNB, potentially explaining why the case described by Borde et al. and our findings differ from the current LNB diagnostics.

The question is why and how neutrophilic granulocytes are involved in the pathophysiology of LNB, since infections with the spirochaete *B. burgdorferi* are quite different from those caused by common bacteria. Instead of triggering an intense immune defence reaction against bacterial surface proteins and lipopolysaccharides, *B. burgdorferi* can hide from the immune system and silently disseminate [24]. It is not yet resolved whether they reach the CNS via the bloodstream or the peripheral nerves. Either way, in patients suffering from LNB, silent dissemination comes to a halt, evidenced by a pronounced intrathecal immune response against the invading spirochete and neurological manifestations. A role for granulocytes as phagocytes is possible, as in vitro studies have shown that granulocytes can take up spirochetes [25]. Notably, the phagocytic activity of granulocytes could be significantly enhanced once the spirochetes are opsonized by specific antibodies, indicating a certain potential to cooperate with B cells and plasma cells [26]. Moreover, the recent detection of elevated levels of CXCL1, a granulocyte-attracting chemokine, in the CSF of patients with definite LNB, allows for an immunological explanation for their presence [27]. A non-human primate model of clinical LNB, furthermore, has revealed a pattern of granulocyte presence in the CSF of rhesus macaques similar to that obtained from our human data—not the dominant cell type, not obligatory in all, but definitely present [28]. An alternative explanation for the intrathecal presence of granulocytes in patients suffering from LNB is an epiphenomenon of Th17-associated immunity. In fact, granulocytes respond to both CXCL1 and CCL20, both of which are also Th17-related markers, with growing evidence supporting the involvement of Th17 cells in the intrathecal immune response of LNB [27]. This includes high levels of Il-17A in the CSF during the acute phase and the delayed recovery from LNB [29], a phenomenon which, again, is in line with our observed association between granulocytes and acuity.

Our study is limited by its retrospective nature and by the fact that routine diagnostic cytological evaluations were performed by different neurologists, thus introducing potential inter-rater variability. Nonetheless, our team of clinicians is experienced and specifically trained in CSF cytology evaluation. Although there may be inaccuracies in the estimation of the number of neutrophilic granulocytes in the CSF, this does not affect their presence or absence. A further limitation is that the time from the symptom onset to the lumbar puncture was patient-reported, meaning it was an estimate. This may explain why our cohort included some cases fulfilling the criteria for acute LNB, including pronounced CXCL13 elevation, despite a reported symptom onset of several months.

To confirm our observations, a prospective multicentric study allowing for a larger cohort of patients with LNB would be desirable. In such a follow-up study, the intrathecal presence of neutrophilic granulocytes should be assessed according to prospectively defined quantification criteria and analyzed by specifically trained CSF cytologists. In addition to the clinical manifestation, age, and gender, in relation to which our study cohort was, most likely, too small to yield useful information, presence of neutrophilic granulocytes should be investigated in association with the clinical course, response to therapy, and potential complications. If confirmed, the occurrence of neutrophilic granulocytes should be integrated into the diagnostic criteria as an acuity marker of LNB. Our observations, therefore, have the potential to increase the value of CSF cytology as a diagnostic tool for LNB beyond allowing early conclusions on the disease etiology. Therefore, rather than being regarded as a possible criterion for ruling out LNB, presence of granulocytes should be included in the diagnostic criteria as a typical sign of LNB, specifically indicating the acute phase.

In conclusion, our study aims to raise awareness of the so-called mixed cell picture featuring neutrophilic granulocytes together with activated mononuclear cells and plasma cells as a typical finding in the context of untreated, acute LNB.

## Figures and Tables

**Table 1:** Patient descriptives.

	Valid	Mean	Median	Min	Max	Std. Dev.
	N					
Age (y)	75	50	59	2	82	24.3
symptom onset to lumbar puncture (d)	74	29	14	1	336	56.8
CSF cell count/μL	75	150	92	8	725	158.6
Borrelia lgG antibody index	66	18	8.3	1.54	177.6	27.2
CSF CXCL13 (pg/mL)	62	440	487	60	710	132.6

CSF: cerebrospinal fluid; CXCL13: Chemokine (C-X-C motif) ligand 13.

**Table 2:** Clinical presentation of patients with definitive LNB.

Clinical Presentation of Patients with Definite LNB	Valid N	Gender (f/m)	Mean Age Range (y)	NO/Few/Several Neutrophilic Granulocytes (N)	Cumulative Presence of Granulocytes (%)
all	75	42/33	59 (2–82)	7/43/25	91
polyradiculitis (+/− facial nerve palsy)	46 (4/42)	26/20	60 (10–82)	5/26/15	89
facial nerve palsy	17	8/9	42 (2–67)	1/11/5	94
meningitis	9	6/3	49 (6–81)	1/4/4	89
others (encephalomyelitis, myelitis, optic neuritis)	3	2/1	45 (15–75)	0/2/1	100

LNB: Lyme neuroborreliosis.

## Data Availability

The datasets used and/or analyzed during the current study are available from the corresponding author upon reasonable request.
